# When the Last Line Fails: Characterization of Colistin-Resistant *Acinetobacter baumannii* Reveals High Virulence and Limited Clonal Dissemination in Greek Hospitals

**DOI:** 10.3390/pathogens14080730

**Published:** 2025-07-24

**Authors:** Dimitrios Karakalpakidis, Theofilos Papadopoulos, Michalis Paraskeva, Michaela-Eftychia Tsitlakidou, Eleni Vagdatli, Helen Katsifa, Apostolos Beloukas, Charalampos Kotzamanidis, Christine Kottaridi

**Affiliations:** 1 Department of Genetics, Development and Molecular Biology, School of Biology, Aristotle University of Thessaloniki, 54124 Thessaloniki, Greece; dimkarakal@gmail.com (D.K.); pmichalis@bio.auth.gr (M.P.); michaelatsitlakidou@gmail.com (M.-E.T.); 2 Veterinary Research Institute Hellenic Agricultural Organization DIMITRA NAGREF Campus, 57001 Thermi, Greece; kotzam@elgo.gr; 3 Laboratory of Biopathology, General Hospital Hippokration, 54642 Thessaloniki, Greece; evagdatli@gmail.com; 4 Microbiology Department, Papanikolaou General Hospital, Exochi, 57010 Thessaloniki, Greece; elen190766@gmail.com; 5 Department of Biomedical Sciences, University of West Attica, 12243 Athens, Greece; abeloukas@uniwa.gr

**Keywords:** *Acinetobacter baumannii*, colistin resistant, *mcr* genes, virulence genes, PFGE

## Abstract

*Acinetobacter baumannii* has emerged as a major pathogen responsible for healthcare-associated infections, particularly in intensive care units, contributing to significant morbidity and mortality due to its multidrug resistance and ability to persist in clinical environments. This study aimed to investigate the phenotypic and genomic characteristics of all multidrug-resistant *A. baumannii* isolates collected between January and June 2022 from two tertiary care hospitals in Thessaloniki, Greece. A total of 40 isolates were included. All isolates exhibited resistance to colistin; however, none harbored the *mcr*-1 to *mcr*-9 genes, as confirmed by polymerase chain reaction (PCR). PCR-based screening for virulence-associated genes revealed high prevalence rates of *basD* (100%), *pld* (95%), *csuE* (87.5%), and *bap* (77.5%). In contrast, *ompA* and *pglC* were not detected. Twitching motility ranged from 2 to 50 mm, with 25% of the isolates classified as non-motile and 20% as highly motile. Swarming motility was observed in all strains. Additionally, all isolates demonstrated positive α-hemolysis, suggesting a potential virulence mechanism involving tissue damage and iron acquisition. Pulsed-field gel electrophoresis (PFGE) revealed significant genomic diversity among the isolates, indicating a low likelihood of patient-to-patient or clonal transmission within the hospital setting. These findings highlight the complex relationship between antimicrobial resistance and virulence in clinical *A. baumannii* isolates and emphasize the urgent need for robust infection control strategies and continued microbiological surveillance.

## 1. Introduction

*A. baumannii* is increasingly acknowledged as a critically important Gram-negative pathogen due to its prevalence and multidrug resistance in contemporary clinical environments. The capacity to persist under harsh environmental conditions and its rapidly evolving resistance profile make it particularly challenging to manage. Although traditionally labeled as non-motile, accumulating evidence now highlights a range of alternative strategies that contribute to its survival and dissemination in healthcare environments. *A. baumannii* is clinically associated with a wide range of refractory infections, notably ventilator-associated pneumonia (VAP), bloodstream infections, meningitis, and wound infections [1,2]. Given these characteristics, it is unsurprising that both the World Health Organization (WHO) and the Centers for Disease Control and Prevention (CDC) have designated *A. baumannii* as a critical priority pathogen for the development of new antimicrobial agents [3]. 

Patients are particularly vulnerable to *A. baumannii* infections—especially multidrug-resistant and colistin-resistant strains—due to several key risk factors. As highlighted by Vrysis et al. [4], prior use of broad-spectrum antibiotics, especially carbapenems, plays a major role in resistance development. Additional risks include ICU-related interventions like mechanical ventilation, prolonged hospitalization, and the use of invasive devices such as urinary or central venous catheters. High illness severity, indicated by scores like APACHE II and SOFA, further increases susceptibility. These factors underscore the need for strict antimicrobial stewardship, careful use of invasive procedures, and robust infection control to prevent resistant *A. baumannii* infections.

In countries like Greece, where our study was conducted, resistance rates to key antimicrobials such as carbapenems and polymyxins are alarmingly high, often surpassing 50% [5]. Colistin has served as a last-line therapeutic option for infections caused by multidrug-resistant (MDR) *A. baumannii*. However, the recent emergence of colistin resistance poses a significant clinical challenge. The underlying resistance mechanisms are primarily chromosomal in nature and involve structural modifications or complete loss of lipopolysaccharides (LPSs), which disrupt colistin’s interaction with the bacterial outer membrane [6,7,8]. In parallel, the identification of plasmid-mediated colistin resistance (*mcr*) genes has introduced a concerning dimension to the threat, as these elements facilitate horizontal gene transfer and the rapid dissemination of resistance across bacterial populations. Ten *mcr* families (*mcr*-1 to *mcr*-10) have been identified globally, mostly in *Escherichia coli* but also in *A. baumannii* strains, with *mcr*-1 and *mcr*-4.3 being the most reported [9,10,11,12]. Although the clinical impact of *mcr* genes is still under investigation, their presence underscores the need for rigorous molecular surveillance to monitor their dissemination and potential contribution to treatment failure [13,14,15]. Beyond resistance, *A. baumannii* exploits many virulent factors that enhance its biological resistance. Biofilm formation is one of the most critical features contributing to chronic infections and treatment failure. The process includes adhesion to surfaces, microcolony development, and production of a protective extracellular matrix, largely driven by quorum sensing [16,17,18,19]. 

Several genes have been associated with biofilm formation and pathogenicity in *A. baumannii.* The *bap* gene encodes a biofilm-associated protein essential for biofilm maturation and stability, while *csuE* is involved in pili assembly, which is necessary for the initial attachment to surfaces. Outer membrane proteins such as ompA and omp33–36 contribute to adhesion, host cell invasion, and immune evasion. The *bauA* and *basD* genes are part of the acinetobactin-mediated iron uptake system, which is crucial for bacterial survival in iron-limited host environments. The *pld* gene encodes phospholipase D, a virulence factor primarily associated with phospholipid degradation, tissue damage, and immune evasion. Although not directly implicated in biofilm regulation, it may support persistence under hostile conditions. Additionally, the *pglC* gene is involved in capsule biosynthesis, which may contribute to immune evasion and environmental resilience, though its direct role in biofilm development remains uncertain [11,12,13,14,20,21,22,23,24,25]. Some bacteria, such as *A. baumannii*, have developed specialized motility mechanisms that enhance their survival and pathogenicity in various environments. Through specialized structures, *A. baumannii* moves on solid and liquid surfaces using swarming, sliding, and twitching motions [26]. Although previously considered non-motile due to the absence of flagella [27], recent studies have shown that it uses type IV pili, with the *pil*A gene playing an important role in their assembly, for twitching motility [16,28,29,30], while surface-associated motility depends on 1,3-diaminopropane (1,3-DAP) and does not require flagella. These mechanisms contribute to bacterial dissemination and evasion of the host immune system [16,28,31]. Additionally, hemolytic activity enables the release of iron and other nutrients from host cells, further supporting bacterial growth in vivo [26,32,33]. 

Molecular epidemiology tools such as PFGE are commonly employed to assess the clonal relatedness of clinical *A. baumannii* isolates and trace the spread of MDR clones within healthcare settings [34]. Given the clinical significance and epidemiological complexity of MDR *A. baumannii*, continuous monitoring of both resistance mechanisms and virulence profiles is essential to guide effective infection control policies.

The aim of this study was to investigate and characterize all colistin and multidrug-resistant *A. baumannii* strains isolated over a six-month period through the hospital surveillance system from two tertiary care hospitals in Thessaloniki, Greece, during 2022. This analysis sought to better understand their resistance mechanisms, virulence traits, and genetic relatedness in order to inform and guide appropriate infection control measures if needed.

## 2. Materials and Methods

### 2.1. Sampling and Selection Process

The prospective study was conducted in two tertiary hospitals in Thessaloniki, Greece. Data were collected from the laboratory’s database to ensure the accuracy and completeness of the analysis.

For the purposes of this study, two tertiary care hospitals located in Thessaloniki, Greece, were selected for sampling: Hippokration General Hospital, a major public healthcare facility with approximately 900 inpatient beds (hospital A), and General Hospital G. Papanikolaou, which has a capacity of approximately 750 beds (hospital B). This study was approved by the Institutional Medical Scientific Council of the hospitals (approval protocol number of Hippokration General Hospital: 9336/24-2-2022; approval protocol number of Papanikolaou General Hospital: 557/14-4-2022). Multidrug-resistant *A. baumannii* (MDRAB) isolates were obtained according to the following inclusion criteria: (i) isolates recovered from hospitalized patients between 1 January and 30 June 2022; (ii) isolates collected from all hospital wards; (iii) only one isolate per patient was included, specifically the first MDRAB isolate identified during the hospitalization period; (iv) only *A. baumannii* isolates exhibiting a multidrug-resistant phenotype and resistance to colistin.

### 2.2. Antimicrobial Susceptibility Testing 

Antimicrobial susceptibility testing was performed in duplicate using the Biomerieux VITEK 2 microbial identification system (bioMérieux, Marcy l’Étoile, France). Colistin susceptibility was further performed using the microdilution method (microdilution colistin broth sensitivity). Colistin susceptibility was also confirmed by the quantitative MIC determination method using the e-test from Lioflichem (Lioflichem MIC test strip, Roseto degli Abruzzi, Italy) [35,36,37]. 

The results were interpreted according to the European Committee on Antimicrobial Susceptibility Testing (EUCAST) guidelines. Multidrug-resistance (MDR), extensive drug resistance (XDR), and pandrug resistance (PDR) were defined as previously proposed [38]. 

### 2.3. Detection of Colistin Resistance Genes

Multidrug-resistant *A. baumannii* isolates were screened for the presence of colistin resistance genes (*mcr*-1 to *mcr*-9) using polymerase chain reaction (PCR) assays. The protocols performed were described by Rebelo et al. [39], while for *mcr*-6 to *mcr*-9, detection was performed as described by Borowiak et al. [40]. For the detection of *mcr* genes, we employed well-characterized control strains: *mcr*-1 (*E. coli* 2012-60-1176-27), *mcr*-2 (*E. coli* KP27), *mcr*-3 (*E. coli* 2013-SQ352), *mcr*-4 (*E. coli* DH5α), and *mcr*-5 (*Salmonella* 13-SA01718). The primers used for PCR are listed in Table 1.

### 2.4. Detection of Virulence Genes

PCR was employed to screen the presence of eight genes implicated in virulence, namely, *bap*, *ompA*, *csuE*, *bauA*, *basD*, *omp33–36*, *pld*, and *pglC*. Detection of *bap*, *ompA*, *csuE*, and *bauA* genes was performed according to the protocols described by Sharma et al. [41], while amplification of *basD*, *omp33–36*, *pld*, and *pglC* genes followed the procedures outlined by Alanazi et al. [42] (Table 1).

### 2.5. Assessment of Biofilm-Formation Ability

The ability of MDRAB strains to generate biofilm was evaluated using the crystal staining technique described by O’Toole [43] and using 96-well microtiter plates. MDRAB strains were grown o/n (overnight) in LB (Luria–Bertani) medium with a shaking speed of 200 rpm at 37 °C. The OD600 was measured to determine the density of the bacterial cells in each culture. Briefly, each well received 180 μL of soybean tryptic broth (TSB) (Merck KGaA, Darmstadt, Germany; Cat. No. T8907), with the addition of 2% glucose (*w*/*v*) and 20 μL of the bacterial liquid culture taken directly from the overnight cultures. The plates were then kept at 37 °C for 72 h, washed three times with water for injection (WFI), and dried at 37 °C for about half an hour. Afterwards, 200 µL of crystalline violet was applied to each well, and after washing, 200 μL of 95% ethanol was added. The ability for biofilm formation was assessed by measuring the optical density at 595 nm (OD595) using a multispectral reader (Thermo Labsystems MS352). TSB (Merck KGaA, Darmstadt, Germany; Cat. No. T8907) enriched with 2% (*w*/*v*) glucose was used as a negative control. The cut-off value for the optical density (ODc) was defined as three standard deviations above the mean OD of the negative control. Depending on the resulting OD values, microbial strains were classified as no (N) biofilm formers (OD < ODc), weak (W) biofilm formers (ODc < OD ≤ 2 × ODc), moderate (M) biofilm formers (2 × ODc < OD ≤ 4 × ODC), or strong (S) biofilm formers (4 × ODc < OD), according to the criteria defined by Borges et al. [44]. Two *S. aureus* isolates, previously classified as moderate and strong biofilm formers [45], were also used to verify the test. All tests were performed three times, and the results of the microtiter plate tests were averaged.

### 2.6. Detection of Hemolytic Activity

The hemolytic activity of the isolated strains was evaluated following the protocol of Boone et al. [26] with minor modifications. Initially, the strains were cultured on Columbia Agar plates supplemented with 5% sheep blood (Col+S+SB PLUS, bioMérieux) and incubated for 24 h at 37 °C. Subsequently, 2–3 isolated colonies were subcultured in 5 mL of Luria–Bertani (LB) broth (Merck KGaA, Darmstadt, Germany; Cat. No. L3522) and incubated overnight at 37 °C. On the following day, the bacterial suspension was adjusted to a 0.5 McFarland standard, and 3 μL of this suspension was inoculated onto fresh Columbia Agar plates supplemented with 5% sheep blood. The plates were incubated for 24 h at 37 °C and then examined for the presence of β-hemolysis around the colonies [26].

### 2.7. Detection of Twitching and Swarming Motility

Twitching motility was assessed using LB agar plates containing 1% agar. A fresh colony from each isolate was inoculated vertically into the agar with a sterile toothpick, reaching the bottom of the plate. The plates were incubated at 37 °C for 48 h. After incubation, the agar was carefully removed, and the bottom of the plate was stained with 1% crystal violet. Bacterial spread was observed macroscopically, and motility was assessed by measuring bacterial spreading in millimeters (mm). Swarming motility was evaluated on LB agar plates with 0.4% agar. Each plate was inoculated on the surface with 0.2 μL of overnight liquid culture and incubated closed at 37 °C for 48 h. The diameter of the spreading zone was recorded in millimeters, and as a quantitative measure, motility was assessed by measuring bacterial spreading in millimeters (mm). Twitching motility was categorized based on the diameter of the spreading zone as follows: non-motile (−) isolates: <5 mm; moderately (+) motile: 5–20 mm; and highly (++) motile: >20 mm. Swarming motility was considered positive (+) when the spreading exceeded 10 mm from the point of inoculation [45,46].

### 2.8. Pulsed-Field Gel Electrophoresis (PFGE)

PFGE, using the restriction enzyme ApaI, was performed for the MDRAB isolates according to a protocol suggested by Seifert et al. [47]. 

Salmonella enterica serotype Branderup strain H9812, digested with 40 units of XbaI (New England Biolabs, Beverly, MA, USA), was used as a size standard. Electrophoresis conditions, using the CHEF-DR III system (Bio-Rad Laboratories Inc., Hercules, CA, USA), were 14 °C for 19 h, with pulse times ranging from 5 to 20 s at an angle of 120°, and the voltage was 6 V cm^−1^. Gels were stained with ethidium bromide (1 μg/mL) and photographed. A database containing all *Apa*I PFGE patterns was created using the Bionumerics software (ver.6.6 Applied Maths, Sint-Martens-Latem, Belgium). A dendrogram was constructed using Dice’s similarity coefficient and the unweighted pair group method with arithmetic mean (UPGMA), with 1.5% of tolerance and optimization. Clusters were defined using a genetic similarity threshold of 85%, with the additional requirement that each cluster comprised at least three isolates.

## 3. Results

### 3.1. Isolate Collection

A total of 40 MDRAB isolates were obtained, each from a distinct hospitalized patient. Of these, 27 isolates were collected from Hippokration Hospital (designated as A) and 13 from Papanikolaou Hospital (designated as B), both of which are tertiary care hospitals in Thessaloniki. Most isolates were recovered from patients in the Intensive Care Unit (ICU, n = 19), followed by the Internal Medicine Unit (IMU, n = 7), the Respiratory Failure Unit (RDU, n = 3), and the Plastic Surgery Unit (PSU, n = 3), while the remaining 8 originated from various other hospital departments. The patient population consisted of 23 males and 17 females.

### 3.2. Antimicrobial Susceptibility Testing

Overall, 38 out of the 40 *A. baumannii* isolates (95%) were classified as PDR, while 2 isolates were classified as XDR. The susceptibility results to colistin, as determined by both the reference microdilution method and the e-test method, showed that all isolates exhibited resistance to colistin, with a minimum inhibitory concentration (MIC) of ≥4 μg/mL. These two methods were fully consistent with the resistance profile to colistin as determined by the BioMérieux VITEK 2 system of the two hospitals (Table S1).

### 3.3. Molecular Detection of Colistin Resistance and Virulence-Associated Genes

**Figure 1:**
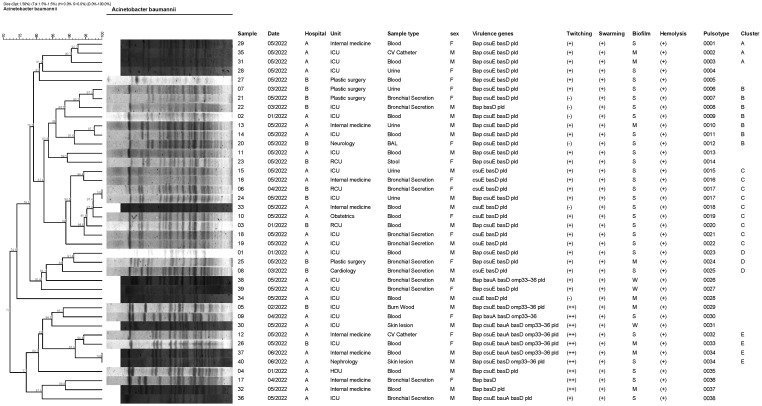
PFGE dendrogram of 40 multidrug-resistant *A. baumannii* isolates recovered from two tertiary care hospitals in Thessaloniki, Greece (2022).

Analyses of the samples using multiplex PCR did not reveal the presence of any *mcr*-1, *mcr*-2, *mcr*-3, *mcr*-4, *mcr*-5, *mcr*-6, *mcr*-7, *mcr*-8, or *mcr*-9 genes in any of the examined *A. baumannii* strains (Figure 1).

Regarding the virulence-associated genes, the most frequently detected were *basD*, which was present in all 40 isolates (100%), and *pld*, found in 38 isolates (95%). The *csuE* gene was identified in 35 isolates (87.5%), while *bap* was present in 31 (77.5%). In contrast, the *omp33–36* gene was detected in only eight isolates (20%), and *bauA* was detected in seven isolates (17.5%). None of the isolates tested positive for *ompA* or *pglC* (Figure 1, Table S1). 

A limitation of the present study was the absence of positive control strains in the PCR assays targeting *mcr* 6–9, as well as virulence genes. Despite this limitation, all reactions were performed using rigorously optimized conditions based on established protocols from the literature.

### 3.4. Phenotypic Characterization of Biofilm Formation, Hemolysis, and Motility

Among the total isolates analyzed (n = 40), 67.5% (n = 27) exhibited a strong biofilm-forming ability (strongly positive, S), 22.5% (n = 9) were classified as positive (moderate biofilm formers, M), and 10% (n = 4) were categorized as weakly positive (weak biofilm formers, W) (Figure 1). 

A clear hemolysis zone surrounding the bacterial colonies was observed in all cases, indicating positive (+) α-hemolytic activity in 100% of the isolates. 

The twitching motility assay revealed that 10 isolates (25%) were categorized as non-motile (−), 22 (55%) as moderately (+) motile, and 8 (20%) as highly (++) motile in the twitching motility assay. The twitching diameters ranged from 2 mm to 50 mm, with a mean value of 10.55 mm, indicating considerable phenotypic variability. 

In contrast, all isolates exhibited positive (+) swarming motility, with spreading zones ranging from 11 mm to 30 mm and a mean diameter of 19.7 mm. Most of the isolates derived from skin lesions, catheter tips, and urine cultures displayed high twitching activity (≥30 mm), whereas those isolated from blood and respiratory specimens demonstrated heterogeneous motility profiles displaying either non-motile or moderately motile behavior. The highest twitching value observed (50 mm) was found in an isolate from a skin lesion (Figure 1, Table S1).

### 3.5. Genetic Diversity of Isolates

Among the 40 *A. baumannii* isolates analyzed by PFGE, 38 distinct pulsotypes (designated 0001–0038) were identified. At an 85% similarity cut-off, five major clusters (A–E) were defined. Cluster A included three isolates, cluster B included seven isolates, cluster C included nine isolates, cluster D included three isolates, and cluster E included four isolates. Cluster A was exclusively found in Hospital A, whereas isolates belonging to clusters B, C, D, and E were distributed across both hospitals (Figure 1).

## 4. Discussion

In this study, we analyzed 40 MDRAB isolates from two tertiary care hospitals in Greece. The majority exhibited a PDR phenotype and universal resistance to colistin. No *mcr* genes were detected, while key virulence-associated genes, including *basD*, *pld*, *csuE*, and *bap*, were prevalent. Most isolates demonstrated strong biofilm formation, α-hemolytic activity, and swarming motility, with considerable variability in twitching motility. PFGE analysis revealed high genetic diversity, with 38 distinct pulsotypes, while five major clusters were identified at an 85% similarity threshold. 

The mounting threat of colistin-resistant *A. baumannii* is increasingly evident in recent regional and international reports, which collectively emphasize the urgent need for coordinated surveillance and intervention. While Ozoaduche et al. [48] did not directly assess colistin resistance, their identification of high-risk clones such as ST2 in both clinical and environmental settings in the Western Balkans and Hungary suggests the potential for the silent dissemination of resistant strains beyond hospital walls, particularly in regions with environmental antibiotic pressure from veterinary use. This concern is reinforced by Bouali et al. [49], who documented clinical isolates in Algeria harboring both carbapenemase genes and phenotypic resistance to colistin, illustrating a troubling convergence of resistance determinants within hospital-associated strains. Similarly, Salem et al. [50] demonstrated the widespread mobilization of resistance elements via transposons and plasmids in Egyptian hospitals, confirming the genomic plasticity that enables rapid acquisition and dissemination of resistance traits, including those to colistin. The outbreak described by Hidalgo et al. [51] in a Spanish burn unit—where ST1 isolates co-producing NDM-1 and OXA-23 also exhibited resistance to colistin and cefiderocol—further highlights the risk of pan-resistant lineages emerging under intense antimicrobial pressure.

The results of the present study highlight the alarming extent of multidrug resistance in clinical isolates of *A. baumannii*, with 95% of strains showing resistance to all antimicrobials tested. This observation is consistent with previous reports from Greece and other countries, including carbapenems and colistin [52,53,54]. Of particular interest is the fact that in our study, the 40 MDRAB strains from two tertiary hospitals in Thessaloniki did not present any of the *mcr* resistance genes (*mcr*-1 to *mcr*-9), which are usually associated with colistin resistance in Gram-negative bacteria. To date, *mcr*-mediated colistin resistance has not been reported in *A. baumannii* in Greece, rendering this resistance mechanism currently absent or extremely rare. This finding aligns with recent clinical studies reporting phenotypic colistin resistance in *A. baumannii* despite the absence of *mcr* genes, suggesting that the underlying mechanisms may involve chromosomal mutations in regulatory loci such as *pmrAB* or *lpxA*, *lpxC*, and *lpxD* [55,56,57]. These alternative mechanisms are increasingly recognized as major contributors to colistin resistance in clinical environments. Notably, studies including Greek isolates have documented mutations in the *pmrAB* two-component regulatory system and *lpx* lipid A biosynthesis genes associated with colistin resistance. Palmieri et al. [58] identified the *pmrB* A226V mutation in all colistin-resistant isolates examined between 2015 and 2017, alongside additional *pmrA/pmrB* substitutions linked to elevated MICs and lipid A modifications. Similarly, Oikonomou et al. [59] reported mutations in both the *pmrCAB* operon and *lpxACD* genes in resistant clinical isolates from central Greece, implicating these changes in reduced colistin susceptibility. Experiments aimed at further elucidating the underlying resistance mechanisms—specifically whole-gene sequencing and analysis of these chromosomal loci—are currently underway by our research team for the *A. baumanni* strains studied in the present work.

The genetic analysis of virulence factors showed that the *bap* gene, associated with mature biofilm stabilization, was present in the majority of the isolates, supporting its established role in persistence and virulence [60,61]. Most of the isolates classified as strong biofilm formers carried the *bap* gene, suggesting a correlation between *bap* presence and high biofilm ability. Furthermore, the presence of other virulence genes, such as *csuE*, *basD*, and *pld*, underlines the multidimensional mechanisms by which *A. baumannii* can evade host defenses and survive in the hospital environment. In particular, the presence of the *pld* gene was significantly associated with strong biofilm production. Although *pld* is primarily involved in phospholipid degradation and immune evasion [24,25], its correlation with biofilm-forming abilities suggests a complementary role in virulence expression, possibly enhancing colonization and persistence in clinical settings. The high prevalence of *basD*, *pld*, and *bap* in *A. baumannii* isolates suggests a multifactorial virulence strategy, where these genes may act synergistically to enhance colonization, persistence, and pathogenicity. *basD*, a key gene in acinetobactin biosynthesis, facilitates iron acquisition in iron-limited environments such as the human host, supporting bacterial survival and metabolic activity. *pld* encodes phospholipase D, which contributes to epithelial damage and may aid in host tissue invasion, potentially complementing *bap*-mediated biofilm formation, which enhances surface adherence, immune evasion, and antibiotic tolerance. The co-occurrence of these genes may therefore reflect a coordinated virulence strategy where nutrient acquisition (*basD*), tissue invasion (*pld*), and persistence (*bap*) reinforce one another, particularly in chronic or device-associated infections. This functional interplay underscores the importance of targeting multiple virulence determinants in anti-virulence therapy development. The absence of the *ompA* and *pglC* genes in our strains, however, suggests that these virulence factors may not be critical for strains circulating in this geographic region. Similar absence patterns have been observed in other regional studies, possibly reflecting clonal or environmental adaptation [62]. 

In terms of pathogenicity, our study revealed that a significant proportion of MDRAB strains displayed the ability to form biofilms, which is a key factor in their persistence and resistance to antimicrobial therapies. Most of the isolates were classified as strongly positive for biofilm formation, which is consistent with previous studies that have highlighted the role of biofilm in *A. baumannii* pathogenicity. This observation agrees with recent findings that link strong biofilm formation with increased resistance to antimicrobials and host immune evasion [42]. 

In our study, all isolates demonstrated hemolytic activity, suggesting that this trait may play a consistent role in *A. baumannii* pathogenicity. The ability to induce hemolysis could facilitate tissue damage and aid in evasion of the host immune response, potentially enhancing the organism’s capacity to establish and sustain infections. This finding agrees with recent studies that describe α-hemolysis as a conserved virulence trait in MDRAB, contributing to host cell lysis and immune modulation [63].

Swarming motility was detected in all strains, indicating that this form of motility may represent a stable phenotype in clinical MDRAB strains. In contrast, twitching motility showed significant phenotypic heterogeneity. High twitching values were more frequently associated with strains derived from skin lesions or catheters, which may be related to the adaptation of the microorganism to surface environments. These findings suggest the possible involvement of motility in both colonization and biofilm development. These observations are consistent with the current literature, which supports the role of motility in facilitating both colonization and biofilm development in *A. baumannii* [28,29].

The PFGE analysis of 40 multidrug-resistant *A. baumannii* isolates revealed striking genetic diversity, identifying 38 distinct pulsotypes. Despite this diversity, five primary clusters emerged. Cluster A, consisting of three isolates, was confined to Hospital A, indicating a possible localized clonal expansion. In contrast, clusters B through E (comprising 7, 9, 3, and 4 isolates, respectively) were distributed across both hospitals, pointing toward inter-facility circulation—a pattern consistent with findings from other surveillance studies. Overall, the high genetic heterogeneity observed suggests that the dissemination of *A. baumannii* in our setting is more likely driven by sporadic introductions from environmental reservoirs, community sources, or patients’ endogenous flora, rather than sustained clonal spread within a single institution. 

Similar PFGE-based investigations across Greek healthcare settings have reported both transient clonal expansions and high strain turnover. For instance, a 2009 study at Papageorgiou Hospital in Thessaloniki demonstrated the rise and fall of dominant clones over time, suggesting dynamic replacement rather than long-term endemic persistence [64]. In an earlier multicenter study (1998–1999) involving nine Athens ICUs, 68% of isolates clustered into two major clonal groups, indicating widespread inter-hospital dissemination [65]. Conversely, a decade-long molecular epidemiology study across eight Greek hospitals (2000–2009) reported an increase in clonal diversity and emergence of novel sequence types, reflecting ongoing diversification [66]. Our observation of numerous unique strains interspersed with small clusters aligns with this pattern, suggesting a mixed epidemiological scenario. The presence of clusters across both hospitals may reflect shared reservoirs, patient transfers, or environmental persistence, while the predominance of unique pulsotypes supports repeated independent introductions. This contrasts with classical outbreak dynamics, where large, homogeneous PFGE clusters suggest extensive cross-transmission, such as in documented *A. baumannii* epidemics in surgical ICUs [67]. Collectively, our findings emphasize the complex epidemiology of *A. baumannii* in the hospital environment and support the need for surveillance strategies tailored not only to outbreak containment but also to managing ongoing, multifocal introductions.

## 5. Conclusions

This study underscores the growing threat of multidrug-resistant *A. baumannii* in clinical environments. Its resistance to multiple antimicrobial classes—including, in some instances, colistin combined with its capacity for biofilm formation and a wide array of virulence factors—reinforces its role as a significant agent of hospital-acquired infections. Although *mcr* genes were not detected, the presence of alternative resistance mechanisms requires further investigation. The pronounced genetic heterogeneity observed via PFGE suggests that direct patient-to-patient transmission is limited, with sporadic introductions from environmental reservoirs likely playing a more prominent role. To mitigate this challenge, continuous molecular surveillance and strengthened infection control strategies are crucial. In particular, targeted measures such as biofilm-disrupting disinfectants, monitoring for motility and biofilm-associated strains, and customized cleaning protocols in high-risk areas may help reduce transmission and infection rates.

## Figures and Tables

**Table 1:** Primers used in the present study.

Target Gene		Primer Sequence (5′ → 3′)	Amplicon Size (bp)	References
*mcr-*1	FW	AGTCCGTTTGTTCTTGTGGC	320	[39]
	RV	AGATCCTTGGTCTCGGCTTG		
*mcr*-2	FW	AAGTGTGTTGGTCGCAGTT-3′	715	[39]
	RV	TCTAGCCCGACAAGCATACC-3′		
*mcr*-3	FW	AAATAAAAATTGTTCCGCTTATG	929	[39]
	RV	AATGGAGATCCCCGTTTTT		
*mcr*-4	FW	TCACTTTCATCACTGCGTTG	1116	[39]
	RV	TTGGTCCATGACTACCAATG		
*mcr*-5	FW	ATGCGGTTGTCTGCATTTATC	1644	[39]
	RV	TCATTGTGGTTGTCCTTTTCTG		
*mcr*-6	FW	AGCTATGTCAATCCCGTGAT	252	[40]
	RV	ATTGGCTAGGTTGTCAATC		
*mcr*-7	FW	GCCCTTCTTTTCGTTGTT	551	[40]
	RV	GGTTGGTCTCTTTCTCGT		
*mcr*-8	FW	TCAACAATTCTACAAAGCGTG	856	[40]
	RV	AATGCTGCGCGAATGAAG		
*mcr*-9	FW	TTCCCTTTGTTCTGGTTG	1011	[40]
	RV	GCAGGTAATAAGTCGGTC		
*csuE*	FW	TCAGACCGGAGAAAAACTTAACG	320	[41]
	RV	GCCGGAAGCCGTATGTAGAA		
*bap*	FW	AATGCACCGGTACTTGATCC	715	[41]
	RV	TATTGCCTGCAGGGTCAGTT		
*ompA*	FW	ATGAAAAAGACAGCTATCGCGATTGCA	929	[41]
	RV	CACCAAAAGCACCAGCGCCCAGTTG		
*bauA*	FW	ACCACTTGCACCGTTGGTAT	1644	[41]
	RV	GCAAGTTGCAACATCGAGCA		
*basD*	FW	CTCTTGCATGGCAACACCAC	252	[42]
	RV	CCAACGAGACCGCTTATGGT		
*omp33–36*	FW	ATTAGCCATGACCGGTGCTC	551	[42]
	RV	CCACCCCAAACATGGTCGTA		
*pglC*	FW	TGGATGAGTTAGCTGC	856	[42]
	RV	TTTTACAAATAGTTAAGC		
*pld*	FW	CCGTCAATTACGCCAAGCTG	1011	[42]
	RV	CTGACGCTACCTGACGGTTT		

## Data Availability

Data is contained within the article.

## Supplementary Materials

The following supporting information can be downloaded at: https://www.mdpi.com/article/10.3390/pathogens14080730/s1, Table S1: Phenotypic and genotypic characteristics of *A. baumannii* isolates.
