# Supplementary material for: When the Last Line Fails: Characterization of Colistin-Resistant Acinetobacter baumannii Reveals High Virulence and Limited Clonal Dissemination in Greek Hospitals
Source: Pathogens. 2025 Jul 24;14(8):730. doi: 10.3390/pathogens14080730 (PMC12389286; doi:10.3390/pathogens14080730)
Supplement: Supplementary file 1 [file pathogens-14-00730-s001.zip › pathogens-3753582-supplementary.pdf]

SOLATES	Antimicrobial resistance profile																														Virulence Genes (%)									Motility		
	AMX	AMK	AMP	ATM	FEP	CFM	CTX	FOX	CPT	CRO	CXM	CXMA	CHL	CIP	COL	DOX	ETP	FOS	GEN	IPM	LVX	MEM	MXF	NIT	TZP	TET	TGC	TOB	SXT	*mcr 1-9* (0)	*Bap* (77.5)	*ompA* (0)	*csuE* (87.5)	*bauA* (17.5)	*basD* (100)	*omp33–36* (20)	*Pld* (95)	*pglC* (0)	Biofilm % (SF 67.5, MF 22.5, WF 22.5)	Swarming motility % (P 100)	Twitching Motility % (HM 20, MM 55, NM 25)	Hemolysis-*α* % (P 100)
1	R	R	R	R	R	R	R	R	R	R	R	R	R	R	R	R	R	R	R	R	R	R	R	R	R	R	R	R	R	N	P	P	P	N	P	N	P	P	SF	P	MM	P
2	R	R	R	R	R	R	R	R	R	R	R	R	R	R	R	R	R	R	R	R	R	R	R	R	R	R	R	R	R	N	P	N	P	N	P	N	P	N	SF	P	NM	P
3	R	R	R	R	R	R	R	R	R	R	R	R	R	R	R	R	R	R	R	R	R	R	R	R	R	R	R	R	R	N	P	N	P	N	P	N	P	N	SF	P	MM	P
4	R	R	R	R	R	R	R	R	R	R	R	R	R	R	R	R	R	R	R	R	R	R	R	R	R	R	R	R	R	N	P	N	P	N	P	N	P	N	SF	P	HM	P
5	R	R	R	R	R	R	R	R	R	R	R	R	R	R	R	R	R	R	R	R	R	R	R	R	R	R	R	R	R	N	P	N	P	N	P	P	P	N	MF	P	HM	P
6	R	R	R	R	R	R	R	R	R	R	R	R	R	R	R	R	R	R	R	R	R	R	R	R	R	R	R	R	R	N	N	N	P	N	P	N	P	N	SF	P	MM	P
7	R	R	R	R	R	R	R	R	R	R	R	R	R	R	R	R	R	R	R	R	R	R	R	R	R	R	R	R	R	N	P	N	P	N	P	N	P	N	SF	P	MM	P
8	R	R	R	R	R	R	R	R	R	R	R	R	R	R	R	R	R	R	R	R	R	R	R	R	R	R	R	R	R	N	N	N	P	N	P	N	P	N	SF	P	MM	P
9	R	R	R	R	R	R	R	R	R	R	R	R	R	R	R	R	R	R	R	R	R	R	R	R	R	R	S	R	R	N	P	N	N	P	P	P	N	N	SF	P	HM	P
10	R	R	R	R	R	R	R	R	R	R	R	R	R	R	R	R	R	R	R	R	R	R	R	R	R	R	R	R	R	N	N	N	P	N	P	N	P	N	SF	P	MM	P
11	R	R	R	R	R	R	R	R	R	R	R	R	R	R	R	R	R	R	R	R	R	R	R	R	R	R	R	R	R	N	P	N	P	N	P	N	P	N	MF	P	MM	P
12	R	R	R	R	R	R	R	R	R	R	R	R	R	R	R	R	R	R	R	R	R	R	R	R	R	R	R	R	R	N	P	N	P	P	P	P	P	N	SF	P	HM	P
13	R	R	R	R	R	R	R	R	R	R	R	R	R	R	R	R	R	R	R	R	R	R	R	R	R	R	R	R	R	N	P	N	P	N	P	N	P	N	SF	P	MM	P
14	R	R	R	R	R	R	R	R	R	R	R	R	R	R	R	R	R	R	R	R	R	R	R	R	R	R	R	R	R	N	P	N	P	N	P	N	P	N	SF	P	MM	P
15	R	R	R	R	R	R	R	R	R	R	R	R	R	R	R	R	R	R	R	R	R	R	R	R	R	R	R	R	R	N	N	N	P	N	P	N	P	N	SF	P	MM	P
16	R	R	R	R	R	R	R	R	R	R	R	R	R	R	R	R	R	R	R	R	R	R	R	R	R	R	R	R	R	N	N	N	P	N	P	N	P	N	SF	P	MM	P
17	R	R	R	R	R	R	R	R	R	R	R	R	R	R	R	R	R	R	R	R	R	R	R	R	R	R	R	R	R	N	P	N	N	N	P	N	N	N	SF	P	HM	P
18	R	R	R	R	R	R	R	R	R	R	R	R	R	R	R	R	R	R	R	R	R	R	R	R	R	R	R	R	R	N	N	N	P	N	P	N	P	N	SF	P	MM	P
19	R	R	R	R	R	R	R	R	R	R	R	R	R	R	R	R	R	R	R	R	R	R	R	R	R	R	R	R	S	N	N	N	P	N	P	N	P	N	SF	P	MM	P
20	R	R	R	R	R	R	R	R	R	R	R	R	R	R	R	R	R	R	R	R	R	R	R	R	R	R	R	R	R	N	P	N	P	N	P	N	P	N	SF	P	NM	P
21	R	R	R	R	R	R	R	R	R	R	R	R	R	R	R	R	R	R	R	R	R	R	R	R	R	R	R	R	R	N	P	N	P	N	P	N	P	N	SF	P	MM	P
22	R	R	R	R	R	R	R	R	R	R	R	R	R	R	R	R	R	R	R	R	R	R	R	R	R	R	R	R	R	N	P	N	N	N	P	N	P	N	SF	P	NM	P
23	R	R	R	R	R	R	R	R	R	R	R	R	R	R	R	R	R	R	R	R	R	R	R	R	R	R	R	R	R	N	P	N	P	N	P	N	P	N	WF	P	MM	P
24	R	R	R	R	R	R	R	R	R	R	R	R	R	R	R	R	R	R	R	R	R	R	R	R	R	R	R	R	R	N	P	N	P	N	P	N	P	N	SF	P	MM	P
25	R	R	R	R	R	R	R	R	R	R	R	R	R	R	R	R	R	R	R	R	R	R	R	R	R	R	R	R	R	N	P	N	P	N	P	N	P	N	MF	P	MM	P

**26**	R	R	R	R	R	R	R	R	R	R	R	R	R	R	R	R	R	R	R	R	R	R	R	R	R	R	R	R	R	N	P	N	P	P	P	P	P	N	MF	P	HM	P
**27**	R	R	R	R	R	R	R	R	R	R	R	R	R	R	R	R	R	R	R	R	R	R	R	R	R	R	R	R	R	N	P	N	P	N	P	N	P	N	SF	P	N M	P
**28**	R	R	R	R	R	R	R	R	R	R	R	R	R	R	R	R	R	R	R	R	R	R	R	R	R	R	R	R	R	N	P	N	P	N	P	N	P	N	SF	P	MM	P
**29**	R	R	R	R	R	R	R	R	R	R	R	R	R	R	R	R	R	R	R	R	R	R	R	R	R	R	R	R	R	N	P	N	P	N	P	N	P	N	SF	P	MM	P
**30**	R	R	R	R	R	R	R	R	R	R	R	R	R	R	R	R	R	R	R	R	R	R	R	R	R	R	R	R	R	N	P	N	P	P	P	P	P	N	W F	P	HM	P
**31**	R	R	R	R	R	R	R	R	R	R	R	R	R	R	R	R	R	R	R	R	R	R	R	R	R	R	R	R	R	N	P	N	P	N	P	N	P	N	MF	P	N M	P
**32**	R	R	R	R	R	R	R	R	R	R	R	R	R	R	R	R	R	R	R	R	R	R	R	R	R	R	R	R	R	N	P	N	N	N	P	N	P	N	MF	P	HM	P
**33**	R	R	R	R	R	R	R	R	R	R	R	R	R	R	R	R	R	R	R	R	R	R	R	R	R	R	R	R	R	N	N	N	P	N	P	N	P	N	SF	P	N M	P
**34**	R	R	R	R	R	R	R	R	R	R	R	R	R	R	R	R	R	R	R	R	R	R	R	R	R	R	R	R	R	N	N	N	P	N	P	N	P	N	MF	P	N M	P
**35**	R	R	R	R	R	R	R	R	R	R	R	R	R	R	R	R	R	R	R	R	R	R	R	R	R	R	R	R	R	N	P	N	P	N	P	N	P	N	MF	P	MM	P
**36**	R	R	R	R	R	R	R	R	R	R	R	R	R	R	R	R	R	R	R	R	R	R	R	R	R	R	R	R	R	N	P	N	P	P	P	N	P	N	SF	P	MM	P
**37**	R	R	R	R	R	R	R	R	R	R	R	R	R	R	R	R	R	R	R	R	R	R	R	R	R	R	R	R	R	N	P	N	P	P	P	P	P	N	MF	P	HM	P
**38**	R	R	R	R	R	R	R	R	R	R	R	R	R	R	R	R	R	R	R	R	R	R	R	R	R	R	R	R	R	N	P	N	N	P	P	P	P	N	W F	P	MM	P
**39**	R	R	R	R	R	R	R	R	R	R	R	R	R	R	R	R	R	R	R	R	R	R	R	R	R	R	R	R	R	N	P	N	P	N	P	N	P	N	W F	P	N M	P
**40**	R	R	R	R	R	R	R	R	R	R	R	R	R	R	R	R	R	R	R	R	R	R	R	R	R	R	R	R	R	N	P	N	P	N	P	P	P	N	SF	P	HM	P

Table S1: Phenotypic and genotypic characteristics of *A.baumannii* isolates

AMX: Amoxicillin, AMK: Amikacin, AMP: Ampicillin, ATM: Aztreonam, FEP: Cefepime, CFM: Cefixime, CTX: Cefotaxime, FOX: Cefoxitin, CPT: Ceftaroline, CRO: Ceftriaxone, CXM: Cefuroxime, CXMA: Cefuroxime Axetil, CHL: Chloramphenicol, CIP: Ciprofloxacin, COL: Colistin, DOX: Doxycycline, ETP: Ertapenem, FOS: Fosfomycin, GEN: Gentamycin, IPM: Imipenem, LVX: Levofloxacin, MEM: Meropenem, MXF: Moxifloxacin, NIT: Nitrofurantoin, TZP: Piperacillin/Tazobactam, TET: Tetracyclin, TGC: Tigecycline, TOB: Tobramycin, SXT: Trimethoprim/Sulfamethoxazole.

R=resistant, S=sensitive, P = positive, N = negative, SF=strong formers, MF=moderate formers, WF=weak formers, HM = highly motile, MM = moderately motile, NM = non-motile
